# High gain low profile horn array with circular polarization using a 3D printed anisotropic dielectric composite material at 38 GHz

**DOI:** 10.1038/s41598-022-23441-0

**Published:** 2022-11-08

**Authors:** Nelson Castro, Francisco Pizarro, Eva Rajo-Iglesias

**Affiliations:** 1 Department of Signal Theory and Communication, University Carlos III of Madrid, 28911 Madrid, Spain; 2 Pontificia Universidad Católica de Valparaíso, Escuela de Ingeniería Eléctrica, Valparaiso, Chile

**Keywords:** Engineering, Electrical and electronic engineering

## Abstract

In this work, the design of a fully 3D-printed dielectric polarizer based on anisotropic engineered material operating at 38 GHz is presented. The anisotropy conditions to obtain circular polarization are achieved by using an array of dielectric strips, manufactured using two different commercially available filaments for 3D-printing. To illuminate the polarizer, a low-profile horn linear array fed by transverse slots is designed and manufactured. The results show good agreement between simulations and measurements, with the designed polarizer covering the whole operation band of the antenna by keeping a similar gain when compared to the structure without the polarizer.

## Introduction

In order to satisfy the higher demand of bandwidth in wireless communications, the new communication systems are migrating to the upper part of the electromagnetic wave spectrum, known as millimeter wave band^1^. In particular, and after several studies of channel characterization^2^, the frequency of 38 GHz is one of the candidate frequencies for 5G devices within this part of the spectrum. One drawback of increasing the operational frequency for the communication systems is that propagation losses increase, and therefore there is a need to implement solutions that can compensate this issue, for instance by increasing the directivity of the radiating elements. To this aim, solutions such as antenna arrays and lens antennas have been proposed in the literature^3,4^. One particular solution that can be used with the objective of being integrated into base stations where directional antennas are needed, is the use of waveguide slot arrays^5^. These topologies, in general terms, can be divided into two possible designs, the longitudinal slot array and the transverse slot array^6^. In particular, transverse slot arrays are much less used due to the required element spacing that is around one guided wavelength, which is always larger than the free space wavelength, and therefore, produces grating lobes^7^. The use of this transverse slot array as feeding structure for low-profile horns has proved to be of interest, as the use of these horns that are directive radiating elements produces the suppression of the grating lobes in around 12 dB compared to the main lobe^8^. This design is suitable for 5G communication systems due to its low-losses and low profile.

Other common solution used to improve the performance of high frequency systems is the use of circularly polarized antennas. Circular polarization (CP) presents several advantages compared to linear polarized antennas. To name a few, the delay spread can be reduced and this ensures higher levels of received power compared to linear polarization^9,10^, and also the CP is more robust than linear polarization^11^. There are different methods to achieve CP on the antennas that involve either the feeding network, such as dual feed feeding structures^12^ or changes in the antenna geometry^13^. However, these implementations can be difficult to implement when using aperture or waveguide-based antennas due to the manufacturing issues or limitations on the standard array feeding networks. One solution to overcome these issues is the use of an external structure that can polarize the impinging electromagnetic wave coming from the antenna^14^. This can be accomplished using the anisotropic characteristics of artificial materials designed for this purpose^15^. In this context the use of metasurfaces constitute an excellent solution^16,17^, in adition with the use periodic arrays of metallic layer with fixed unitcells^18,19^ show very good performance with low losses due to the fully metallic approach with the main drawback being the complexity of the design, in general bulky structures and the cost of fabrication.

The use of dielectric materials has some advantages compared to these metallic designs due to an easy integration and manufacture together with the addition of a much lighter weight which can be beneficial for some applications. The possibility of using fast prototyping techniques such as 3D printing allows to design new composite materials suitable for linear to circular polarizers. One way to have a dielectric anisotropic composite material is by using periodic dielectric strips with two different dielectric constants^20^. This technique uses alternating strips with a high contrast of relative permittivity, which leads to an effective permittivity depending on the polarization of the incident electric field. The resulting effective dielectric constants introduce a phase shift between electric field components, which can lead to a CP. This technique was previously used integrated in a modified Fresnel lens antenna to obtain CP^21^ and also, by itself as circular polarizer for a horn antenna^22^, showing good results. However in the latter work there is no analysis of the effects on the antenna matching or radiation pattern. Furthermore none of these works study dependence of the CP on the geometry of the polarizer itself, which can be critical due to the interaction of the electric field with the dielectric materials.

Another important issue to be treated is the manufacture of the polarizer, which can be done using additive manufacturing. In recent years, 3D-printing has been largely used for the implementation of different high-frequency devices, thanks to the introduction of low-loss dielectric filaments and highly conductive filaments. These new materials, together with the reduction of the price of high-precision 3D-printers make possible the manufacture of structures that were either too difficult or expensive to implement^23–26^. In particular, for the polarizer, it can open the possibility of a wide range of implementations that can be adapted to different structures in terms of conformity and needed dielectric constants. This manufacturing approach has been explored at the mm-wave frequency range such as in Refs.^27,28^ using adoc printing methods. In Ref.^29^ the fabrication using fused filament deposition is explored at the 24 GHz frequency band with very good results in terms of bandwidth and axial ratio. In this work, this approach is taken further in the mm-wave frequency bands with the design and manufacturing of a circular polarizer for a low profile horn array feed by transverse slots at 38 GHz, fabricated using low cost and low loss 3D printing at mm-wave frequencies.

## Design of the linear antenna array

**Figure 1:**
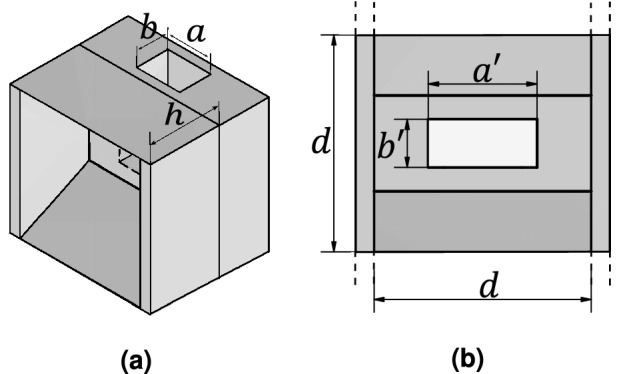
Low profile pyramidal horn where $a = 4.88$ mm, $b = 3.556$ mm, $a' = 3.75$ mm, $b' = 1.2$ mm, $h = 7.79$ mm and $d = 13.57$ mm (**a**) Isometric view (**b**) Front view.

**Figure 2:**
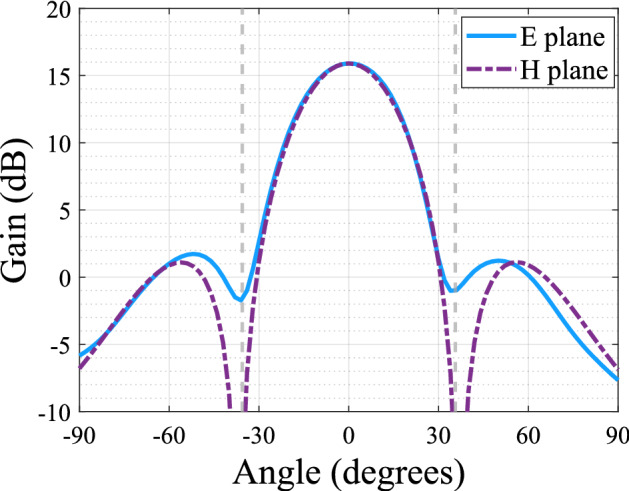
Gain radiation pattern of the low profile horn antenna.

The first step is to design the linear antenna array used as feeding structure for the dielectric anisotropic polarizer. To this aim, a single low-profile pyramidal horn has to be first designed and optimized following some guidelines from Refs.^30,31^. The objective is to have a high-aperture efficiency in each single element of the array by exciting two modes of the horn. The proposed horn has to be placed in the wide side of a rectangular waveguide with the transverse slots, with the constrain that the aperture dimension in one of the sides should be around one guided wavelength to have the maximum of radiation at broadside. Due to the configuration of the array, grating lobes are expected, but they will be attenuated by the radiation pattern of the horn antenna. The position of the grating lobes is calculated using Eq. (1), which is a function of the element spacing and the free space wavelength. The proposed horn is presented in Fig. 1. After optimization, the antenna aperture is set to $d = 13.57$ mm with a height of $h = 7.79$ mm. The dimensions of the transverse slot are initially obtained using the transmission line theory where $a'$ and $b'$ are set to 3.75 mm and 1.2 mm respectively, however the final values for $a'$ and $b'$ will be discussed in the following section, due to the influence of the polarizer on these dimensions. It is important to mention that in order to implement the proposed low-profile horn into the array, it is necessary to use a waveguide with cross section $a = 4.88$ mm and $b = 3.556$ mm, which is not a standard one. This waveguide provides the required guided wavelength at 38 GHz. The simulated gain radiation pattern is presented in Fig. 2, and it shows a maximum gain of 15.9 dB with an obtained aperture efficiency of 95%. The attenuation at $\pm 35.6^{\circ }$ which is the calculated position for the grating lobes, for the E plane is of 17.5 dB. This means that when implemented into the array, the attenuation of the grating lobes is expected to be around this value.

1 $$\begin{aligned} \theta _{GL} = sin^{-1}\frac{m\lambda }{d}, \quad m = \pm 1, \pm 2, \pm 3. \end{aligned}$$

**Figure 3:**
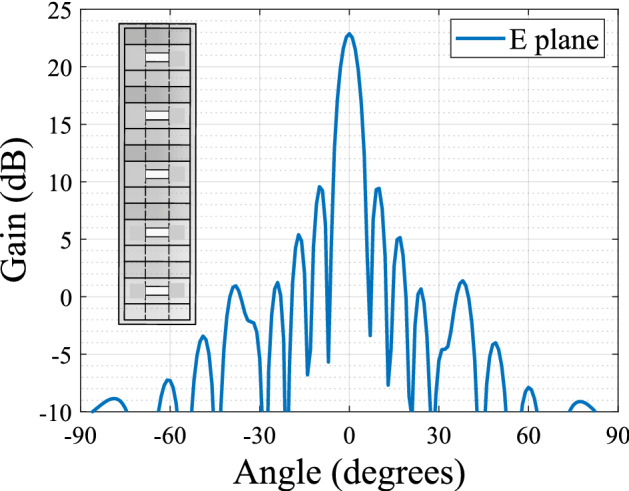
E plane radiation pattern of the 5 element array and array topology.

The proposed horn is then implemented in the array where the slots to feed them are spaced by a distance of $d = 13.57$ mm. Five horns are placed as it can be seen in Fig. 3 where the radiation pattern of the array is also presented.

## Design of the anisotropic polarizer and array integration

**Figure 4:**
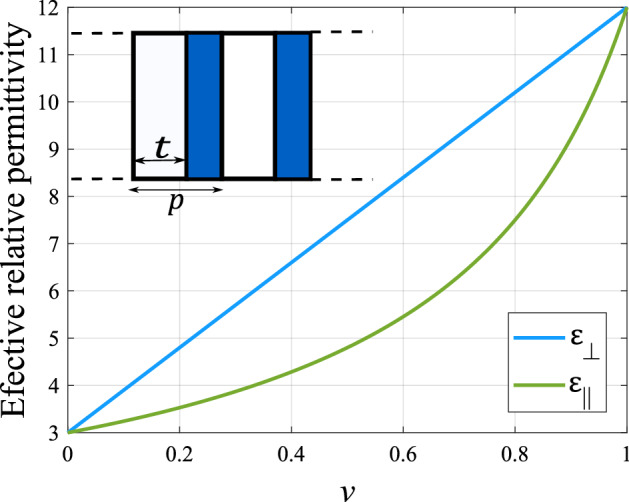
Effective permittivity of the composite material as a function of the volume fraction of the high-permittivity region of the polarizer.

The design of the anisotropic dielectric polarizer consists of a material composed of periodic arrangement of dielectric strips with two different dielectric constants leading to an anisotropic behavior. The effective relative permittivity of the composite material will depend on the electric field polarization of the incident electromagnetic wave. In this analysis, the incident wave into the polarizer has two electric field components, $E_{\perp }$ and $E_{||}$ for perpendicular and parallel polarization w.r.t. the strips, with the same amplitude and phase. For this field, each component propagating in the material will have different phase velocities, and if the thickness is correctly chosen, the phase difference between the components at the output will be 90 degrees leading to circular polarization. Regarding the anisotropic dielectric material, it is composed of a relatively high-permittivity section with $\varepsilon _h$ and a relatively low permittivity section with $\varepsilon _l$. The value of the permittivity will be a function of the volume fraction of the material which is a ratio between the area of the high permittivity material *t* with respect to the strips period *p* as shown in Fig. 4. The values that this effective permittivity can take, named as $\varepsilon _{\perp }$ and $\varepsilon _{||}$, are defined by the lower and upper limits of the permittivity defined by Eqs. (2) and (3), known as absolute Wiener bounds^32^. The nominal values of permittivity used in the design are $\varepsilon _l$ = 3 and $\varepsilon _h$ = 12, corresponding to the values of two ABS filaments for 3D-printing available off-the-shelf^33^. The curve that describes these absolute bounds and the composite material are presented in Fig. 4.

2 $$\begin{aligned}&\varepsilon _{||} = v\varepsilon _h + (1 - v)\varepsilon _l, \end{aligned}$$

3 $$\begin{aligned}&\varepsilon _{\perp } = \frac{\varepsilon _h\varepsilon _l }{v\varepsilon _l + (1-v)\varepsilon _h}. \end{aligned}$$

The proposed dielectric polarizer is designed to have a rectangular shape in order to ease the integration with the array. The volume fraction of the relatively high permittivity material is $v = 0.55$, and the periodicity of the dielectric strips is $p = 2$ mm, resulting in the values of permittivity $\varepsilon _{||}$ = 7.95 and $\varepsilon _{\perp }$ = 5.106. To get 90$^\circ$ of phase shift the thickness *l* must be calculated using Eqs. (4) and (5), where $\tau _{1,2}$ and $\rho _{1,2}$ are the transmission and reflection coefficients in the first and second interfaces of the dielectric slab calculated for the parallel or perpendicular polarization, and $\beta _{||,\perp }$ is the phase constant of the dielectric material also for each polarization. This lead to a fixed thickness of $l = 3.4$ mm.

4 $$\begin{aligned}&T_{||} = \frac{\tau _{1_{||}}\tau _{2_{||}}e^{-j\beta _{||}l}}{1+\rho _{1_{||}}\rho _{2_{||}}e^{-2j\beta _{||}l}}, \end{aligned}$$

5 $$\begin{aligned}&T_{\perp } = \frac{\tau _{1_{\perp }}\tau _{2_{\perp }}e^{-j\beta _{\perp }l}}{1+\rho _{1_{\perp }}\rho _{2_{\perp }}e^{-2j\beta _{\perp }l}}. \end{aligned}$$

**Figure 5:**
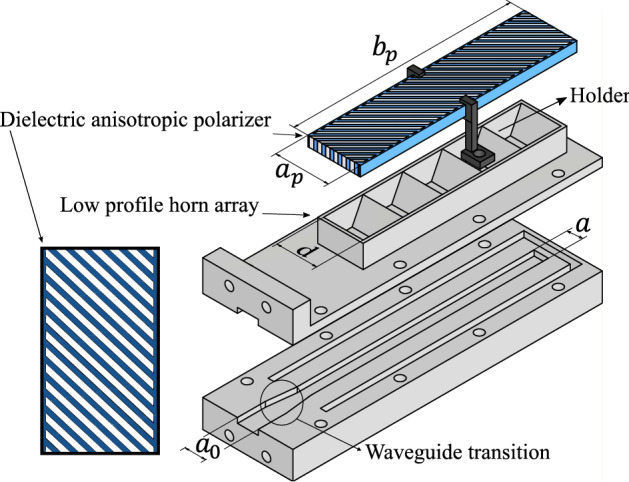
Exploded view of the dielectric polarizer and the transverse horn array where $a_0$ = 7.112 mm, *a* = 4.88 mm, *b* = 3.556 mm, *d* = 13.57 mm, $a_p$ = 18 mm and $b_p$ = 74.2 mm.

**Figure 6:**
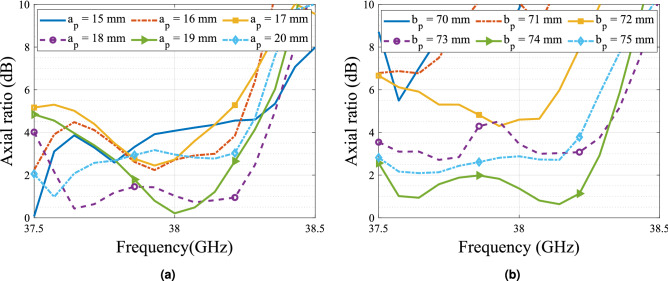
Parametric study simulation results. (**a**) Variation of a$_p$ when b$_p$ = 74.2 mm. (**b**) Variation of b$_p$ when a$_p$ = 18 mm.

As the antenna array is operating using a non-standard waveguide, a waveguide to waveguide transition is implemented. This transition ensures the impedance transition from a commercially available WR-28 with cross section $a_{0} = 7.112$ mm and $b = 3.556$ mm to the custom waveguide with $a = 4.88$ mm and $b = 3.556$ mm. In Fig. 5 the array and the polarizer are presented, a dielectric holder to attach the polarizer to the antenna array is also considered. This holder will be manufactured with 3D printing with a filament of relative permittivity 2.51. The dielectric strips in the polarizer are rotated 45$^\circ$ with respect to the polarization of the electric field of the impinging wave in order to force the decomposition of the vector to obtain the required phase delay between the two components for the given thickness of the material. After optimization with respect to the resulting axial ratio, the lateral dimensions of the polarizer are set as $a_p$ = 18 mm and $b_p$ = 74.2 mm. These values were obtained after the parametric study which is presented in Fig. 6. The results show a high sensitivity of the axial ratio w.r.t these parameters, which can be problematic in the manufacturing process. In addition, the impedance matching of the antenna must consider the impedance of the polarizer due to the scattering of the wave when facing the dielectric material. To this aim, the slot dimensions are optimized to match the antenna at 38 GHz, being finally set as $a' = 4.5$ mm and $b' = 1.5$ mm.

**Figure 7:**
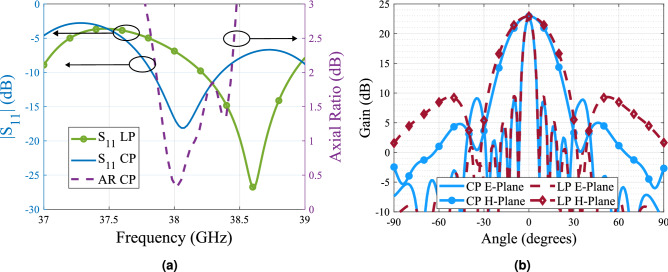
Simulation results of the array for Linear polarization (LP) and circular polarization (CP). (**a**) Reflection coefficient $S_{11}$ in dB and axial ratio in dB as function of the frequency. (**b**) E-Plane and H-Plane gain radiation pattern.

Due to the high sensitivity of the polarizer to the changes of its lateral dimensions, a small dielectric edge of $\varepsilon _r$ = 3 of 0.5 mm is added to the structure as a way to ease the manufacturing process. The simulation of the structure with this edge is presented in Fig. 7 with the same lateral dimensions given in the previous paragraph. The simulation results show a bandwidth of 750 MHz for a less than 3 dB axial ratio and a good impedance matching of the antenna. In terms of radiation pattern, the maximum gain is not affected by the use of the polarizer, while the grating lobes are bigger than expected due to unwanted scattering produced by the polarizer. However, these values remains under the 12 dB of attenuation considered in most of the previous works regarding this type of antenna^8,31^.

### 3D printing manufacturing and measurements

**Figure 8:**
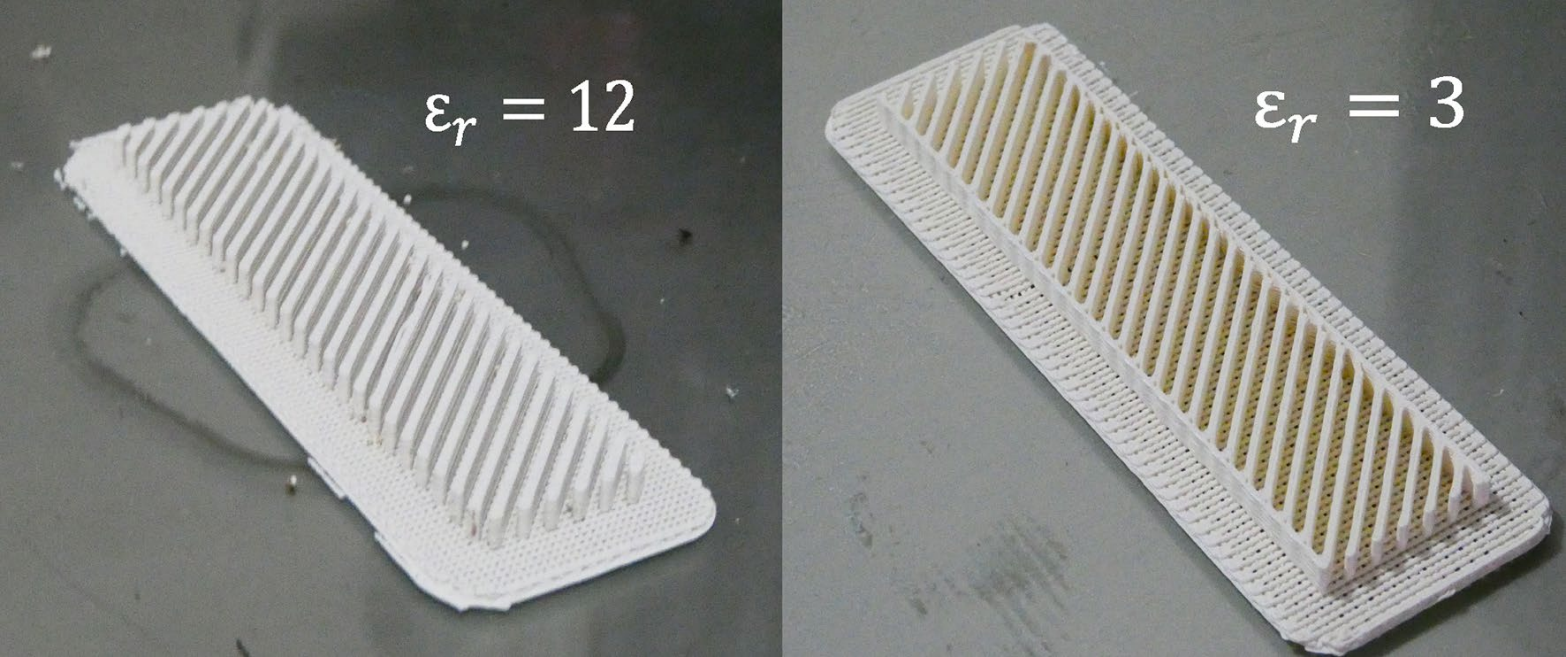
3D-printed parts of the dielectric polarizer: high-permittivity part (left) and low permittivity part (right).

**Figure 9:**
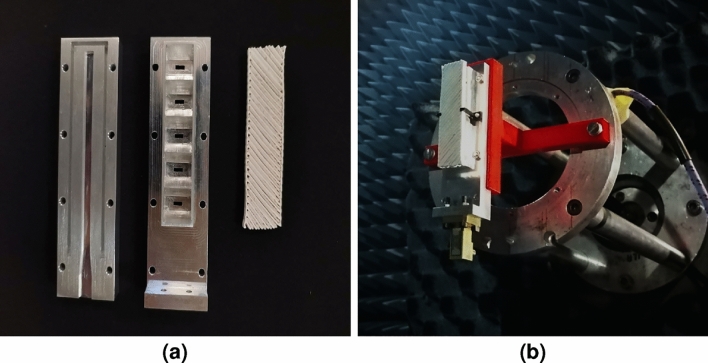
Manufactured prototype of the antenna array and dielectric polarizer. (**a**) De-assembled parts of the antenna (**b**) Assembled antenna at the measuring process.

The proposed dielectric polarizer is manufactured using 3D printing. Two filaments, the ABS300 and the ABS1200, are used for the manufacturing of the composite material. Each part is printed using a 3D printer suitable for high temperature ABS filaments. The parameters used for the printing process are tabulated in Table 1 and the manufactured parts of the polarizer can be seen in Fig. 8. To set the pieces together, both the high and low permittivity parts are glued together. In Fig. 9 the manufactured prototype can be seen, the antenna and polarizer are assembled using a dielectric holder printed in PLA ($\varepsilon _r$ = 2.51) and then measured.

**Table 1:** Printing parameters.

Parameters	ABS300	ABS1200
Printing temperature ($^{\circ }$C)	220	250
Build plate temperature ($^{\circ }$C)	80	100
Print speed (mm/s)	10	5
Wall speed (mm/s)	5	2.5
Flow	140 %	150 %

**Figure 10:**
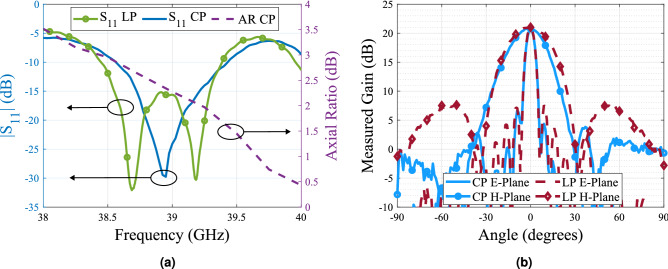
Measurement results of the array with the dielectric polarizer (**a**) Reflection coefficient and axial ratio (**b**) Comparison between the measured radiation pattern with and without the dielectric polarizer.

The magnitude of the axial ratio and the gain radiation pattern are measured in an anechoic chamber operating up to 40 GHz, while the magnitude of the $S_{11}$ is measured using a vector network analyzer (VNA). From the obtained results shown in Fig. 10 it is observed that the antenna is operating 1 GHz above the design frequency. The E-plane and H-plane radiation pattern at 39 GHz is plotted in Fig. 10b for both the antenna with and without the polarizer. The achieved gain in both cases is above 20.4 dBi with grating lobes more than 17 dB below. Regardless the frequency shift, the bandwidth of the antenna being around 850 MHz represents the same percentage with respect to the central frequency compared to the simulations. Moreover, the polarizer does not add any extra losses as it can be seen by the gain measurement where for both cases with polarizer (CP) and without it (LP), the gain at the broadside remains almost constant, having only a 0.3 dB difference between the case with polarizer and the case without the polarizer. This was expected as previous works in the same frequency band reported similar results in terms of losses^4,34^.

**Figure 11:**
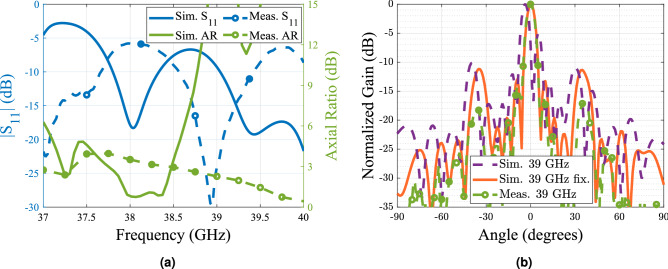
Comparison of the measured and simulated parameters of the antenna (**a**) S$_{11}$ and Axial ratio (**b**) Radiation pattern of the antenna at 39 GHz with two simulation cases; with the original design dimensions reported in this paper and the measured dimensions of the manufactured antenna, compared with the measured radiation pattern of the antenna.

**Figure 12:**
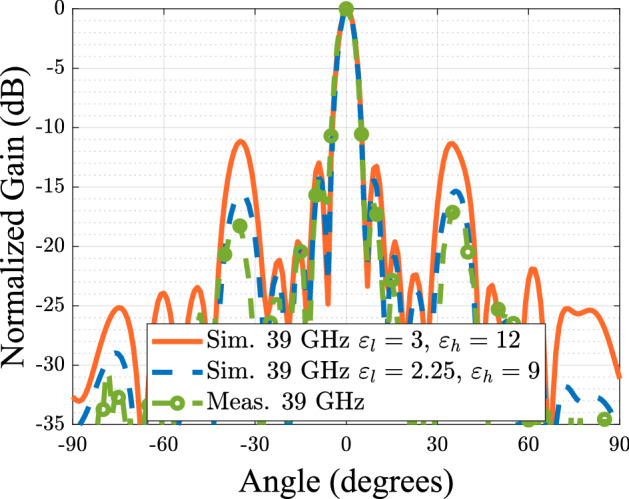
Comparison of the E plane radiation pattern of the antenna with fixed array antenna dimensions and the original values of permittivity with the obtained radiation pattern using permittivities 25% less than the reported values and the measured radiation pattern.

The causes of the frequency shift observed on the measurements can be due to the multiple tolerances regarding the manufacture of the polarizer and the antenna itself. After a careful revision it was seen that the element spacing of the radiating elements is of *d* = 13.44 mm and the waveguide cross section *a* feeding the slots is of 4.68 mm, while the designed values reported in the paper of 13.57 mm and 4.88 mm, respectively. With this change on the dimensions, the spacing between the horns is equivalent to a guided wavelength at 39 GHz, which explaining the broadside radiation at this frequency instead of 38 GHz. In addition imperfections resulting from the gluing process of the high and low permittivity parts lead to small air gaps between the two materials, which can reduce the effective permittivity seen by the wave. To notice that the values given by the manufacturer for the used filaments are measured at 2.4 GHz, therefore any dispersive behavior of the materials at higher frequencies were not considered in the design procedure. In Fig. 11 the simulated radiation pattern with the fixed dimensions and the measured radiation pattern at 39 GHz is presented where good matching between the two cases can be seen. The results shows a much better agreement, however the grating lobe level is still higher than the measured values. This can be explained due to a lower permittivity of the printed parts compared to the one reported of the dielectric filaments. One supposition is that GLL can increase with higher dielectric constants on the polarizer as a consequence of the scattered field in the interface of the device. Therefore as these constants get lower, the GLL will do so. There are some studies such as the reported in Ref.^35^ that states that the permittivity of the printed prototype will be affected by the different parameters set in the slicer software, being overall lower than expected due to the presence of air between layers. As an example of this, a simulation was made with values of 25% less than the nominal values reported for the filaments and compared to the previous results, this is presented in Fig. 12. From the study we can see that as expected, the GLL is reduced and is closer to the measured values.

After the results with the corresponding manufacture tolerance are calculated, an approximation of the measured antenna efficiency is calculated comparing the simulated maximum directivity of the antenna versus the measured values for the cases with and without the polarizer giving as a result an aperture efficiency $\varepsilon _{ap}$ = 69% for the antenna without the polarizer and $\varepsilon _{ap}$ 49% with the polarizer at 39 GHz. This reduction on the antenna efficiency can be explained due to the increase on the effective area of the antenna when the polarizer is included. This means that for the same frequency the maximum directivity will be higher

There are few examples in the literature showing the performance of dielectric 3D printed polarizers operating in the millimeter wave bands. Among them, we can mention^27,28^ in which the polarizers work at 60 GHz with excellent performance in terms of bandwidth and insertion losses, but the manufacturing technology used for the printing is not a standard 3D printing process but a more sophisticated technology and also due to the used materials much more bulky designs relative to the wavelength. The only example where standard 3D printing is used is^29^: in this case, the operation frequency is lower than the one used for our design with the central frequency being 24 GHz. All of these previous examples are more bulky compared to the proposed due to the dielectric constants used in the designs varying from 2.4$\lambda _0$ in Ref.^27^, 1.3$\lambda _0$ of Ref.^28^ and the 0.52$\lambda _0$ thickness presented in Ref.^29^. This last design the more similar to the one presented in this paper with similar results. The main difference is the bandwidth but this is also dependent on the type of antenna used to illuminate the polarizer

## Conclusions

In this work, the use of an anisotropic dielectric composite material as a linear to circular polarizer manufactured using conventional 3D printing technology is presented. The polarizer is implemented in a low-profile high efficiency horn array antenna operating at 38 GHz. This approach is an interesting solution for nowadays communications systems due to the easy integration, fast prototyping and low cost.

The results show that despite the change on the central frequency of the antenna, the polarizer still works properly with no additional losses. This can be assessed by the comparison on the gain radiation pattern of the antenna with and without the polarizer, where the maximum value at the broadside remains constant. The frequency shift can be explained by the unknown dispersive characteristics of the materials used for the polarizer but also by the tolerance of the antenna manufacturing itself which was made by milling. In order to reduce the uncertainties regarding the polarizer manufacture, a proper characterization of the dielectric materials at 38 GHz must be performed and the use of other multi-filament 3D printing techniques can be explored. The possibility to manufacture the polarizer with low-cost 3D-printing can be easily extended to the design of any other type of antenna or any other size of the array.

## Data Availability

The datasets generated during and/or analysed during the current study are available from the corresponding author on reasonable request.
